# Cardiovascular Care of Turner Syndrome Women in Germany: Where Do We Stand?—Results from an Online Patient Survey

**DOI:** 10.3390/healthcare10030504

**Published:** 2022-03-09

**Authors:** Martina Bačová, Pengzhu Li, Leonie Arnold, Robert Dalla-Pozza, Nikolaus Alexander Haas, Felix Sebastian Oberhoffer

**Affiliations:** Division of Pediatric Cardiology and Intensive Care, University Hospital, LMU Munich, 81377 Munich, Germany; martina.bacova@med.uni-muenchen.de (M.B.); pengzhu.li.extern@med.uni-muenchen.de (P.L.); leonie.arnold@med.uni-muenchen.de (L.A.); robert.dallapozza@med.uni-muenchen.de (R.D.-P.); nikolaus.haas@med.uni-muenchen.de (N.A.H.)

**Keywords:** Turner Syndrome, cardiovascular care, Germany

## Abstract

Background: This study aimed to investigate whether the cardiovascular care of Turner Syndrome (TS) women in Germany is in accordance with the latest clinical practice guidelines established by the 2016 Cincinnati international TS meeting. Methods: An anonymous online questionnaire was created to ask TS subjects about existing cardiovascular conditions and cardiovascular care received. Depending on cardiovascular morbidity and type of medical care received, the fulfillment of the latest clinical practice guidelines for the care of TS women was assessed. Results: 120 TS patients were included in this study. The mean age of TS subjects was 36.79 years. Only 80% of subjects received annual blood pressure measurements within the TS cohort, and only 60% received cardiac imaging according to international clinical practice guidelines. More than 55% of TS women did not feel well informed about TS-specific cardiovascular risk factors by their treating physician. Conclusions: A potential lack of cardiovascular care might be present in TS women in Germany as the results of this online patient survey suggest. The cardiovascular care of TS patients, particularly cardiac imaging and patient education, needs to receive more attention.

## 1. Introduction

Turner Syndrome (TS) is a genetic disorder caused by a complete or partial loss of one of the sex chromosomes [1]. Approximately one in 2500 to 3000 female newborns is affected by TS [1]. Besides phenotypical features, such as short stature and gonadal dysgenesis, TS is often accompanied by congenital and acquired cardiovascular diseases [2]. Up to 50% of TS patients display congenital heart defects (CHD), of which the most common forms are bicuspid aortic valve (BAV), coarctation of the aorta (CoA), and partial anomalous venous drainage [2,3]. In addition, TS subjects show an increased prevalence of multiple cardiovascular and cardiometabolic risk factors, such as excess weight, glucose metabolism disorders, lipid metabolism disorders, arterial hypertension, and increased arterial stiffness [2,4,5,6,7]. Furthermore, TS is associated with aortic dilatation, and the risk for acute aortic dissection is considered to be 100-fold higher compared to the general population [2,8]. A national cohort study conducted by Schoemaker et al. revealed that mortality in TS is up to threefold higher compared to the general population and that cardiovascular disease is the most common cause of death [9].

**Figure 1:**
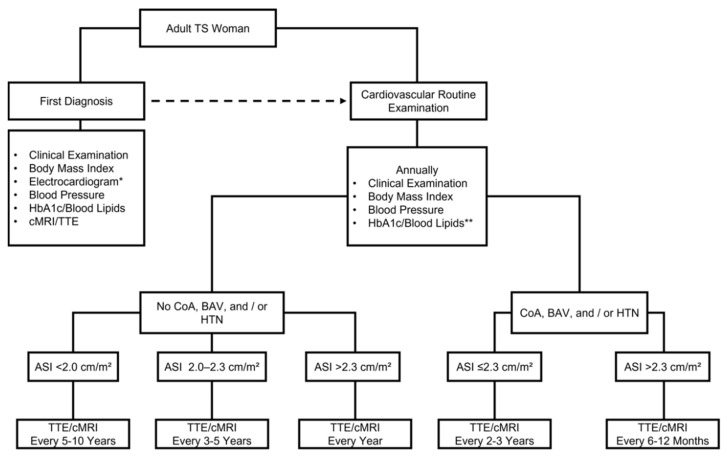
Cardiovascular care of adult women with Turner Syndrome. Modified after the 2016 Clinical Practice Guidelines for the Care of Girls and Women with Turner Syndrome [2]. cMRI, cardiac magnetic resonance imaging. TTE, transthoracic echocardiography. CoA, coarctation of the aorta. BAV, bicuspid aortic valve. HTN, arterial hypertension. ASI, aortic size index. * Hodges formula should be preferred for QTc measurement. If QTc > 460 ms, 24 h Holter-monitoring and exercise testing should be performed for risk stratification. ** Annually if recommended regionally or at least one cardiovascular risk factor is present (arterial hypertension, excess weight, smoking, physical inactivity).

Given the broad spectrum of cardiovascular morbidity, international clinical practice guidelines were established to ensure adequate medical care of TS women [2]. These cardiovascular guidelines are visualized in Figure 1 and can be shortly summarized as follows: 

It is recommended that TS subjects present at specialized multidisciplinary clinics to receive medical care [2]. After the first diagnosis, prompt cardiovascular screening is required. The first cardiovascular screening should include a thorough clinical examination, body mass index (BMI, kg/m^2^) assessment, ambulatory blood pressure measurement, the performance of an electrocardiogram, and the conduction of transthoracic echocardiography (TTE) or cardiac magnetic resonance imaging (cMRI) [2]. Further, HbA1c and blood lipids should be assessed [2]. If QTc is over 460 ms, 24 h Holter-monitoring and exercise testing should be performed for risk stratification [2]. Independent of cardiovascular morbidity, an annual checkup that screens for potential cardiovascular risk factors should be performed in all TS women, as demonstrated in Figure 1 [2]. Depending on the presence of CoA, BAV, arterial hypertension, and aortic size index, TTE or cMRI is recommended to be conducted every six months to 10 years. Nutritional counseling and physical activity encouragement display a crucial aspect in the cardiovascular management of TS women [2,10]. A recent publication of our department revealed that high adherence to the Mediterranean diet tended to be inversely associated with the presence of lipid metabolism disorders in TS women in Germany. Furthermore, failure to achieve ≥600 Metabolic Equivalent-minutes per week doing recreational activities was significantly associated with the presence of arterial hypertension in TS women in Germany [10]. Cardiovascular screening and exercise consultation might be recommended before sports participation to reduce undesirable events due to incorrect engagement [2,10]. 

This study aimed to investigate whether the cardiovascular care of TS women in Germany is in accordance with the latest clinical practice guidelines established by the 2016 Cincinnati international TS meeting [2]. 

## 2. Materials and Methods

### 2.1. Ethical Approval

The Ethics Committee of the Ludwig Maximilians University Munich (Munich, Germany) approved this study on the 7th of May 2021 (project number 21-0403). Prior digital informed consent was obtained from all study participants.

### 2.2. Study Design

The present study design was described in a recent publication of our department [10]: Utilizing the website SurveyMonkey (San Mateo, CA, USA), an anonymous online questionnaire was generated in German (Table S1: Anonymous Online Questionnaire). TS patients were enrolled in cooperation with the German Turner Syndrome Association (Turner-Syndrom-Vereinigung Deutschland e.V.). Moreover, national TS centers, university hospitals, pediatric cardiologists, and cardiologists specialized in treating adults with CHD were notified in writing about the ongoing study. German social media channels concentrating on TS were asked to regularly notify their subscribers about the ongoing questionnaire to guarantee maximum coverage. Study subjects had to digitally confirm the onset of TS and a minimum age of ≥18 years to be qualified for participation. Replies obtained between May 2021 and January 2022 were analyzed for the present study.

### 2.3. Assessment of Patient Characteristics and Cardiovascular Morbidity

The assessment of patient characteristics and cardiovascular morbidity was described in a recent publication of our department [10]: The questionnaire asked for age (years), body height (cm), and body weight (kg). Body mass index (BMI, kg/m^2^) was calculated for each study participant individually. The following weight classifications were used: Underweight if BMI < 18.5 kg/m^2^, normal weight if BMI ≥ 18.5 but <25 kg/m^2^, overweight if BMI ≥ 25 but <30 kg/m^2^ and obese if BMI ≥ 30 kg/m^2^. Histories of arterial hypertension, glucose metabolism disorders, lipid metabolism disorders, smoking, CHD, aortic disease, heart surgery, aortic surgery, and stroke were gathered. TS subjects presenting with arterial hypertension, glucose metabolism disorders, or lipid metabolism disorders were further asked if any medication was taken regularly. Study participants who only partially answered the questions on patient characteristics and/or cardiovascular morbidity were excluded from the analysis.

### 2.4. Assessment of Cardiovascular Care

Questions on cardiovascular care were based on the clinical practice guidelines for the care of girls and women with TS established by the 2016 Cincinnati international TS meeting [2]. TS subjects were asked whether a cardiologist and endocrinologist regularly saw them. Moreover, TS women were asked whether they were treated in a national TS center and/or university hospital and whether they were a member of the German Turner Syndrome Association. Study participants were further questioned about whether a medical professional performed blood drawing and blood pressure measurement within the last 12 months. TS women were further asked whether a 24 h blood pressure monitoring was carried out. In addition, TS women were asked if and when an echocardiographic examination and/or cMRI was conducted last. Study participants could choose one of the following time periods: ≤3 years, >3 but ≤10 years and >10 years. Participating subjects were questioned whether they felt well informed on TS specific cardiovascular risk factors by their treating physician. Furthermore, the effect of the coronavirus pandemic on the medical care received was studied. Study participants who only partially answered the questions on cardiovascular care were excluded from the analysis.

### 2.5. Statistical Analysis

To test for the normal distribution of continuous variables, histograms, QQ-plots, the Kolmogorov–Smirnov test, and the Shapiro–Wilk test were applied. Means and standard deviations were used for all continuous variables. Ordinal and nominal variables are presented as percentages and counts. Data on cardiovascular care was compared between “university hospital/TS center” and “others” using a chi-squared or Fisher’s exact test for expected counts smaller than five. A *p*-value of <0.05 was considered statistically significant. SPSS (IBM SPSS Statistics for Windows, version 26.0. IBM Corp., Armonk, NY, USA) was used for data analysis.

## 3. Results

### 3.1. Patient Characteristics and Cardiovascular Morbidity

In total, 132 TS women participated in the online survey. Due to incomplete responses, only 120 subjects were included in the final analysis. Detailed information on patient characteristics and cardiovascular morbidity are summarized in Table 1.

### 3.2. Cardiovascular Care

#### 3.2.1. General Cardiovascular Care of TS Women in Germany

Table 2 summarizes data on general cardiovascular care of TS women in Germany and informs whether clinical practice guidelines for the care of girls and women with TS established by the 2016 Cincinnati international TS meeting were met [2].

In total, 44.17% of study participants felt well informed on TS-specific cardiovascular risk factors by their treating physician. When asked about the effect of the coronavirus pandemic on the medical care received, 17.5% of TS patients reported that medical care received was negatively affected. Moreover, 78.33% of patients reported no effect of the coronavirus pandemic on the medical care received, and 4.17% stated unknown. 

#### 3.2.2. Cardiovascular Care of TS Women with Arterial Hypertension and/or Congenital Heart Disease in Germany

For the following analysis, 40 TS patients presenting with arterial hypertension and/or CHD were selected. Table 3 summarizes data on the cardiovascular care of these patients and informs whether clinical practice guidelines for the care of girls and women with TS established by the 2016 Cincinnati international TS meeting were met depending on the institution where regular medical care was received [2]. 

#### 3.2.3. Cardiovascular Care of TS Women without Arterial Hypertension and/or Congenital Heart Disease in Germany

For the following analysis, 55 TS patients without arterial hypertension and/or CHD were selected. Table 4 summarizes data on cardiovascular care of these patients and informs whether clinical practice guidelines for the care of girls and women with TS established by the 2016 Cincinnati international TS meeting were met depending on the medical care received [2]. 

#### 3.2.4. Cardiovascular Care of TS Women at Cardiometabolic Risk in Germany 

For the following analysis, 57 TS patients with cardiometabolic risk factors were selected. Cardiometabolic risk factors were defined as overweight, obesity, glucose metabolism disorders and/or lipid metabolism disorders. Table 5 summarizes data on cardiometabolic risk factors of these patients and informs whether clinical practice guidelines for the care of girls and women with TS established by the 2016 Cincinnati international TS meeting were met depending on the institution where regular medical care was received [2]. 

## 4. Discussion

To the best of our knowledge, this is the first study investigating the cardiovascular care of TS women in Germany. An anonymous online questionnaire was used for data acquisition to assess whether the latest clinical practice guidelines established by the 2016 Cincinnati international TS meeting were met [2]. In total, 120 adult TS subjects were enrolled in the present study, making it one of the largest surveys of German-speaking TS women.

### 4.1. Cardiovascular Care of Women with TS in Germany: Where Do We Stand?

The TS cohort studied can be considered relatively young as the mean age was only 36.79 years. Despite the relatively young age, the enrolled TS women displayed distinct cardiovascular morbidity: Over 50% of women demonstrated excess weight, 29.17% reported the presence of arterial hypertension, 18.33% the presence of lipid metabolism disorders, and 7.5% the presence of glucose metabolism disorders. CHD was reported by 20.83% of TS subjects and aortic disease by 13.33%. As some TS women stated unknown for certain cardiovascular diseases, the prevalence’s given in this manuscript might be underestimated (e.g., the prevalence of arterial hypertension and CHD in TS is suggested to be ≥50% in the literature [2,11]). Surprisingly, 14.17% of TS subjects were unknown for aortic disease and 10% for arterial hypertension; both were considered to be major risk factors for increased mortality in TS [9]. Given approximately 80% of TS subjects reported annual blood drawing and blood pressure measurement, and only 60% reported receiving cardiac imaging, as recommended by international guidelines, it can be assumed that a remarkable amount of TS women could potentially be underdiagnosed regarding their cardiovascular morbidity. 

Over 55% of TS subjects did not feel well informed on TS-specific cardiovascular risk factors by their treating physician. As patient education is crucial for compliance and disease prevention, TS women should be extensively informed on cardiovascular risk factors, the importance of healthy lifestyle habits, and the need for regular cardiovascular screening even in the absence of cardiovascular morbidity [2,10]. 

#### 4.1.1. Importance of Specialized Multidisciplinary Clinics in the Cardiovascular Care of TS 

The prevalence of TS is relatively low [1], and not all physicians involved in TS medical care might have extensive knowledge of syndrome-specific cardiovascular morbidity and international care guidelines. It is recommended that TS subjects present at specialized multidisciplinary clinics that offer regular cardiological and endocrinological care [2]. In Germany, university hospitals and national TS centers can be considered as such facilities. This online survey suggests that only 23.33% of TS patients were treated in national TS centers and only 32.5% in university hospitals. Moreover, only 55% of TS subjects reported being regularly seen by a cardiologist and only 60% by an endocrinologist. 

No significant difference in the cardiovascular care of TS women with arterial hypertension and/or CHD was assessed between university hospitals/TS centers and other medical care providers (Table 3). Independent of medical care providers, >90% of hypertensive TS subjects received an annual blood pressure measurement. However, cardiac imaging was not performed in ≥30% of TS patients with arterial hypertension and/or congenital heart disease as recommended by international clinical practice guidelines [2]. Regular cardiac imaging is crucial for risk stratification as these patients are at highest risk for undesired cardiovascular events, such as aortic dissection [2]. 

The cardiovascular care of TS women displaying cardiometabolic risk factors did not differ significantly between university hospitals/TS centers and other medical care providers. Interestingly, TS subjects with lipid metabolism disorders, independent of medical care providers, are rarely reported to be on lipid-lowering medication. 

In TS women without arterial hypertension and CHD, international clinical practice guidelines regarding cardiac imaging tended to be met more often at university hospitals/TS centers compared to other medical care providers [2]. In addition, the number of TS patients who reported having never received cardiac imaging was significantly lower at university hospitals/TS centers than other medical care providers. Interestingly, TS subjects felt significantly better informed on TS-specific cardiovascular risk factors by their treating physician at university hospitals/TS centers compared to other medical care providers. Hence, the data of this study suggest that preventive cardiovascular care and patient education are more prevalent in specialized multidisciplinary clinics, such as university hospitals and national TS centers. 

#### 4.1.2. Potential Influence of the Coronavirus Pandemic 

In this study, the influence of the coronavirus pandemic was assessed. The majority of TS patients (78.33%) reported no effect of the coronavirus pandemic on the medical care received. However, 17.5% of subjects did experience negative effects. Therefore, the results of this study could potentially be altered due to the current pandemic and must be validated in the future. 

### 4.2. Limitations

Limitations of the study design were reported in a previous publication of our department [10]. In this study, 120 TS patients were included, making it one of the largest surveys of German-speaking TS women. However, the relatively small sample size of the current study can be regarded as one limitation. In addition, the demonstrated data must be interpreted with caution due to the subjective nature of the information shared by study participants, as a self-reporting questionnaire was applied. As this study did not ask for zip codes, regional differences in cardiovascular TS care might be present within Germany. As this study only included TS subjects living in Germany, the results might not apply in other countries with different health care systems. A possible impact of genotype-phenotype relationships can be assumed, as this study did not ask for specific TS karyotypes. This study did not ask for the specific type of glucose metabolism disorder, lipid metabolism disorder, CHD, aortic disease, heart surgery, and aortic surgery. Further, the aortic size index was not assessed. Moreover, TS subjects were not asked about the specific type of medication taken or any history of growth hormone or estrogen-progesterone therapy. As this was an online survey for patients only, the questions mentioned above were thought not to be suited to patient medical understanding and thus cause potential data alteration. Hence, the results of this study rather give a first overview of the current cardiovascular care of TS women in Germany. In addition, no data was collected regarding TS knowledge of physicians engaged in the medical care of TS women. 

To analyze the cardiovascular care of TS women in Germany more precisely, national-multicenter studies are required to screen medical records and survey patients and medical professionals in face-to-face interviews. 

## 5. Conclusions

The complex morbidity of TS requires optimal cardiovascular care. This study revealed a potential lack in the current cardiovascular care of TS women in Germany by conducting an online patient survey. Only 60% of study participants received cardiac imaging as recommended by international guidelines. In addition, over 55% of TS subjects did not feel well informed on TS-specific cardiovascular risk factors by their treating physician. The data of this study suggest that preventive cardiovascular care and patient education for TS subjects without arterial hypertension and CHD was more prevalent in specialized multidisciplinary clinics. However, further studies with greater sample size and more objective measures are required to analyze the cardiovascular care of TS women in Germany more precisely. 

## Figures and Tables

**Table 1:** Patient characteristics and cardiovascular morbidity of TS women in Germany.

Patient Characteristics	
Total number	120
Age (years)	36.79 ± 11.90
Height (cm)	153.94 ± 6.88
Body weight (kg)	63.08 ± 15.98
BMI (kg/m^2^)	26.56 ± 6.32
Underweight (%)	7 (5.83)
Normal weight (%)	51 (42.50)
Overweight (%)	35 (29.17)
Obese (%)	27 (22.50)
Arterial hypertension (%)	35 (29.17)
Anti-hypertensive therapy (%)	28 (80.00)
Glucose metabolism disorders (%)	9 (7.5)
Glucose metabolism therapy (%)	9 (100.00)
Lipid metabolism disorders (%)	22 (18.33)
Lipid metabolism therapy (%)	3 (13.64)
Smoker (%)	5 (4.17)
Congenital heart disease (%)	25 (20.83)
Aortic disease (%)	16 (13.33)
Aortic surgery (%)	8 (50)
Cardiac surgery (%)	17 (14.17)
Stroke (%)	3 (2.5)

Mean ± standard deviation are used for normally distributed variables. BMI, body mass index. When asked for the presence of cardiovascular disease, 10% of TS patients stated unknown for arterial hypertension, 5% for glucose metabolism disorders, 13.33% for lipid metabolism disorders, 5.83% for congenital heart disease, and 14.17% for aortic disease.

**Table 2:** General cardiovascular care of TS women in Germany.

Cardiovascular Care	n (%)
Total number	120
Regular care at	
Cardiologist ^a^	66 (55)
Endocrinologist ^a^	72 (60)
TS center	28 (23.33)
University hospital	39 (32.5)
Member of TS Association	44 (36.67)
Blood drawing ≤ 12 months ^a^	98 (81.67)
BP measurement ≤ 12 months ^a^	96 (80)
Cardiac imaging ^a,b^	
Never received	21 (17.5)
Performed as recommended	72 (60)

^a^ Recommendations were based on the clinical practice guidelines for the care of girls and women with TS established by the 2016 Cincinnati international TS meeting [2]. BP, blood pressure. ^b^ Cardiac imaging comprises echocardiographic and/or cardiac magnetic imaging. When asked for regular care, 7.5% of TS patients stated unknown for TS center, and 2.5% of TS patients stated unknown for the University hospital. When asked for the last blood drawing, 2.5% of TS patients stated unknown. When asked for the last BP measurement, 4.17% of TS patients stated unknown. When asked for cardiac imaging, 2.5% of TS patients stated unknown if ever conducted. When asked for the time of last cardiac imaging, 9.17% of TS patients stated unknown.

**Table 3:** Cardiovascular care of TS women with arterial hypertension and/or congenital heart disease in Germany.

Cardiovascular Care	University Hospital/TS Center (n = 18)	Others (n = 22)	*p*-Value
Arterial hypertension (%)	13 (72.22)	15 (68.18)	1
BP measurement ≤ 12 months ^a^ (%)	12 (92.31)	14 (93.33)	1
Anti-hypertensive therapy (%)	12 (92.31)	11 (73.33)	0.33
24h blood pressure monitoring (%)	12 (92.31)	11 (73.33)	0.33
Congenital heart disease (%)	9 (50)	11 (50)	1
Cardiac surgery (%)	5 (55.56)	6 (54.55)	1
Cardiac imaging ^a,b^			
Never received (%)	2 (11.11)	3 (14.29)	1
Performed as recommended (%)	12 (66.67)	14 (70)	1
Well informed on cardiovascular risks factors (%)	9 (52.94)	10 (45.45)	0.75

^a^ Recommendations were based on the clinical practice guidelines for the care of girls and women with TS established by the 2016 Cincinnati international TS meeting [2]. BP, blood pressure. ^b^ Cardiac imaging comprises echocardiographic and/or cardiac magnetic imaging. When asked for cardiac imaging, 1 TS patient in the “Others” group reported unknown if ever conducted. When asked for the time of last cardiac imaging, 2 TS patients in the “Others” group stated unknown. When asked whether the study participants felt well informed on TS-specific cardiovascular risk factors by their treating physician, 1 TS patient in the “University Hospital/ TS Center” group did not answer the question.

**Table 4:** Cardiovascular care of TS women without arterial hypertension and/or congenital heart disease in Germany.

Cardiovascular Care	University Hospital/TS Center (n = 26)	Others (n = 29)	*p*-Value
Blood drawing ≤ 12 months ^a^ (%)	23 (88.46)	23 (79.31)	0.48
BP measurement ≤ 12 months ^a^ (%)	21 (80.77)	20 (74.07)	0.74
Cardiac imaging ^a,b^			
Never received (%)	2 (7.69)	10 (34.48)	0.022 *
Performed as recommended (%)	19 (79.17)	16 (55.17)	0.085
Well informed on cardiovascular risks factors (%)	17 (65.38)	9 (33.33)	0.028 *

^a^ Recommendations were based on the clinical practice guidelines for the care of girls and women with TS established by the 2016 Cincinnati international TS meeting [2]. BP, blood pressure. ^b^ Cardiac imaging comprises echocardiographic and/or cardiac magnetic imaging. When asked for the last BP measurement, 2 TS patients in the “Others” group stated unknown. When asked for the time of last cardiac imaging, 2 TS patients in the “University Hospital/TS Center” group stated unknown. When asked whether the study participants felt well informed on TS specific cardiovascular risk factors by their treating physician, 2 TS patients in the “Others” group did not answer the question. * *p* < 0.05.

**Table 5:** Cardiovascular care of TS women at cardiometabolic risk in Germany.

Cardiovascular Care	University Hospital/TS Center (n = 24)	Others (n = 33)	*p*-Value
Overweight/Obese (%)	17 (70.83)	28 (84.85)	0.32
BP measurement ≤ 12 months ^a^ (%)	12 (70.59)	22 (81.48)	0.47
Glucose metabolism disorders (%)	5 (20.83)	3 (9.1)	0.26
Glucose metabolism therapy (%)	5 (100)	3 (100)	
Blood drawing ≤ 12 months ^a^ (%)	4 (80)	3 (100)	1
Lipid metabolism disorders (%)	8 (33.33)	13 (39.39)	0.78
Lipid metabolism therapy (%)	1 (12.5)	2 (15.38)	1
Blood drawing ≤ 12 months ^a^ (%)	7 (87.5)	12 (92.31)	1
Well informed on cardiovascular risks factors (%)	14 (58.33)	12 (38.71)	0.18

^a^ Recommendations were based on the clinical practice guidelines for the care of girls and women with TS established by the 2016 Cincinnati international TS meeting [2]. BP, blood pressure. When asked for last BP measurement, 1 TS patient in the “Others” group stated unknown. When asked whether the study participants felt well informed on TS specific cardiovascular risk factors by their treating physician, 2 TS patients in the “Others” group did not answer the question.

## Data Availability

The data presented in this study are available upon reasonable request from the corresponding author.

## Supplementary Materials

The following are available online at https://www.mdpi.com/article/10.3390/healthcare10030504/s1, Table S1: Anonymous Online Questionnaire.
