# Supplementary material for: Cardiovascular Care of Turner Syndrome Women in Germany: Where Do We Stand?—Results from an Online Patient Survey
Source: Healthcare (Basel). 2022 Mar 9;10(3):504. doi: 10.3390/healthcare10030504 (PMC8955387; doi:10.3390/healthcare10030504)
Supplement: Supplementary file 1 [file healthcare-10-00504-s001.zip › healthcare-1617916-supplementary.pdf]

**Supplementary file**

**Table S1. Anonymous Online Questionnaire.**

1. How old are you?

    Age in years:

2. What is your body weight?

    Body weight in kilograms:

3. What is your height?

    Height in centimeters:

4. Do you have a glucose metabolism disorder (e.g. diabetes mellitus)?

    Yes

    No

    I do not know

    4.1. Do you have to take medication for your glucose metabolism disorder?

    Yes

    No

5. Do you have a lipid metabolism disorder (e.g. increased cholesterol or triglycerides levels in the blood)?

    Yes

    No

    I do not know

    5.1. Do you have to take medication for your lipid metabolism disorder?

    Yes

    No

6. Do you have high blood pressure?

    Yes

    No

    I do not know

    6.1 Do you have to take medication for your high blood pressure?

    Yes

    No

7. Do you smoke?

    Yes

    No

8. Do you have a congenital heart defect?

Yes
No
I do not know

9. Have you ever had a heart surgery?

Yes
No

10. Do you have an aortic disease (e.g., aortic dilatation, aortic dissection, aortic aneurysm)?

Yes
No
I do not know

11. Have you ever had a surgery of the aorta?

Yes
No
I do not know

12. Have you ever had a stroke?

Yes
No

13. Are you regularly seen by a heart specialist (general adult cardiologist, pediatric cardiologist)?

Yes
No
I do not know

14. Have you ever had a heart ultrasound or an MRI (magnetic resonance imaging) of the heart?

Yes
No
I do not know

14.1 When was the last time you had a heart ultrasound or an MRI (magnetic resonance imaging) of the heart?

≤ 3 years
> 3 years ≤ 10 years
> 10 years
I do not know

15. When was the last time your blood pressure was measured by a medical doctor?

≤ 12 months

> 12 months

I do not know

16. Have you ever had a 24 hour blood pressure monitoring?

Yes

No

I do not know

17. Are you regularly seen by a hormone specialist (endocrinologist)?

Yes

No

I do not know

18. When was the last time you had your blood drawn?

≤ 12 months

> 12 months

I do not know

19. Do you receive regular treatment in a certified "Turner Syndrome Center"?

Yes

No

I do not know

20. Do you receive regular treatment in a university hospital?

Yes

No

I do not know

21. Are you a member of the “German Turner Syndrome Association”?

Yes

No

22. Do you feel well informed on the cardiovascular risk factors of Turner syndrome by your treating physician?

Yes

No

23. Are you not attending or postponing your routine medical examinations due to the current corona pandemic?

Yes

No

I do not know
